# Excessive androgen exposure and risk of malignancies: A cohort study

**DOI:** 10.1111/andr.13648

**Published:** 2024-04-11

**Authors:** Ida M. Heerfordt, Josefine Windfeld‐Mathiasen, Kim Peder Dalhoff, Jon Trærup Andersen, Henrik Horwitz

**Affiliations:** ^1^ Department of Clinical Pharmacology Bispebjerg and Frederiksberg Hospital Copenhagen Denmark; ^2^ Department of Clinical Medicine University of Copenhagen Copenhagen Denmark

**Keywords:** anabolic androgenic steroids, bodybuilding, cancer incidence, cohort study, testosterone

## Abstract

**Background:**

A link between androgen use and the risk of cancers, especially prostate and breast cancer, has been suggested. The knowledge about a possible association is limited.

**Objective:**

The study aimed to investigate cancer incidence rates, particularly those related to prostate and breast cancer, in male androgen users and compare them to a control group.

**Methods:**

We included male androgen users identified through a nationwide anti‐doping testing program in Danish fitness centers from 2006 to 2018. We paired each case with 50 male controls of the same age, selected randomly. The cohort was followed from baseline and until 2023. The outcome was the incidence of prostate cancer, breast cancer, or any cancer excluding non‐melanoma skin cancer.

**Results:**

The study included 1,189 androgen users and 59,450 controls, with a mean age of 27 years at enrolment. During the follow‐up period with a mean length of 11 years, 13 androgen users, and 612 controls were diagnosed with cancer. This resulted in an incidence rate ratio of 1.05 (95% CI: 0.55–1.81). None of the androgen users were diagnosed with prostate or breast cancer.

**Discussion and conclusion:**

Male androgen users did not face an increased short‐term risk of cancer, neither overall nor related to prostate or breast cancer. Our study indicates that the absolute risk of malignancies in androgen users is comparable to that in the background population. However, we cannot exclude androgens as a cancer risk factor due to the limited sample size, relatively short follow‐up period, and subject age.

## 1 INTRODUCTION

Androgenic anabolic steroids, hereafter referred to as androgens, exert a profound impact on the hormonal balance in humans.^1^, ^2^ Bodybuilders using androgens often administer high amounts of testosterone, resulting in blood testosterone levels that exceed the typical physiological range by 5–10 times.^1^, ^3^ High doses of exogenous androgens suppress the normal hypothalamic–pituitary–gonadal axis.^1^, ^3^ Bodybuilders who use these substances can develop low levels of gonadotropins, along with multifold elevated levels of free testosterone and estradiol.^1^, ^3^ Gynecomastia, a frequent issue among bodybuilders using androgens, is likely attributable to elevated levels of estrogen^2^, ^4^ and elevated levels of estrogen have been associated with male breast cancer.^5^, ^6^ Furthermore, some studies suggest a potential association between high levels of free testosterone and an increased risk of prostate cancer^7^, ^8^ just like the antiandrogenic therapy has proven to be an effective treatment for prostate cancer.^9^ Consequently, we hypothesized that the use of androgens could be linked to an elevated risk of developing neoplastic disorders in these two organs. To the best of our knowledge, the risk of malignancies associated with the use of androgens has not previously been investigated in a cohort study.^10^, ^11^

The aim of the current study was to investigate the incidence of cancers with a particular focus on prostate and breast cancer among males who had been sanctioned for androgen use, and to compare their incidence rates with that of a large cohort of age‐matched males from the background population.

## 2 MATERIALS AND METHODS

### 2.1 Design

A matched cohort study.

### 2.2 Outcomes

The primary outcome was the incidence of prostate cancer, breast cancer, or any cancer excluding non‐melanoma skin cancers recorded in the Danish National Patient Register. The secondary outcome was survival following any cancer diagnoses excluding non‐melanoma skin cancers.

### 2.3 Study population

Between January 2006 and March 2018, a total of 342 fitness centers in Denmark partnered with Anti‐Doping Denmark as part of an initiative to promote a safe fitness environment without doping.^2^ Anti‐Doping Denmark, an independent Danish public institution operating under the Ministry of Culture, is dedicated to preventing doping in sports. It conducted around 1,000 annual inspections in fitness centers, as described earlier.^2^, ^12^ The present study included all males who had been expelled from Danish fitness centers as part of the anabolic–androgenic steroid anti‐doping initiative. The expulsions were either due to testing positive for androgens or for declining to submit a sample.^2^ We do not have data on androgen dosage or duration of use. For each case, we randomly selected 50 control individuals who matched in terms of age, sex, and inclusion date using the Danish Civil Registration System.^13^ Participants were tracked until June 2023, or until their date of death or migration.

### 2.4 Registries

All Danish citizens have free and equal access to the national healthcare system. Cancer patients are referred to hospitals where registration of diagnoses (International Classification of Diseases‐10, ICD‐10) in the Danish National Registry of Patients is mandatory.^14^ There is an exception for patients with non‐melanoma skin cancer, a condition often managed by non‐hospital doctors, and therefore, diagnoses for this type of cancer are not consistently registered using ICD‐10 codes.^15^ Consequently, this study does not encompass non‐melanoma skin cancers, specifically those classified under ICD‐10 code C44.

All residents in Denmark have a unique personal identification number, known as the Central Person Register (CPR) number, which serves as an identifier for all interactions with public administration and the healthcare system.^16^ We cross‐referenced the CPR numbers of the study participants with the Danish National Registry of Patients specifically looking for the following ICD‐10 codes:

‐ C00‐C43 and C45‐C97; all cancers except non‐melanoma skin cancers.
‐ C50; malignant neoplasm of breast.
‐ C61; malignant neoplasm of prostate.

Information on survival following cancer diagnosis was obtained from the Danish Civil Registration System.^13^

To characterize the cohorts, all cancers except non‐melanoma skin cancers (ICD‐10 codes: C00‐C43 and C45‐C97) were also identified at baseline. Additionally, the number of cases with gynacomastia (ICD‐10 code: N62), infertility (ICD‐10 code: N46), and testicular dysfunction (ICD‐10 code: E29) at baseline and after follow‐up were obtained.

### 2.5 Statistical analysis

For baseline data, continuous variables were summarized using the mean and standard deviation (SD). The number of cases at baseline was described both in absolute numbers and as a percentage of the population of each respective group. Relative risk, along with its 95% confidence intervals (95% CI), was calculated for comparison.

Incident rates of prostate, breast, or any cancer except non‐melanoma skin cancers during the follow‐up were calculated. Statistics were applied for the Poisson distribution and 95% CI for the incidence rates are presented. The incidence rates of the investigated cancer diagnoses in androgen users were compared with controls and presented as incidence rate ratios (IRR) with exact 95% CI. Survival after cancer diagnoses was analyzed using the Kaplan–Meier estimator and assessed for statistical significance with the log‐rank test using SAS 9.4. The remaining statistics were computed in STATA 17. We used a statistical significance threshold of 0.05.

### 2.6 Ethics

All data were anonymized, and we had no access to any information that could identify individuals. The study received approval from both the Danish Data Protection Agency (BFH‐2017‐105/05949) and the Danish National Board of Health (FSEID‐00003570/FSEID‐00006603). In Denmark, research relying solely on register data is exempt from the need for approval from the Committee on Health Research Ethics.

Due to privacy considerations, events involving fewer than five occurrences are not disclosed, and a detailed list of cancer types is not provided.

## 3 RESULTS

A total of 1,189 doping sanctioned males were included in this study. Our conducted 1:50 sampling resulted in 59,450 male controls. The average age at the time of the doping sentence for cases was 27.4 (SD = 6.9) years, which was matched to the age at enrollment of the control cohort. The cohort characteristics are displayed in Table 1 and provide data regarding the demographics and health profiles of androgen users in contrast to controls.

**TABLE 1:** Cohort characteristics.

Category	Androgen users, *n* = 1,189	Controls, *n* = 59,450	Relative risk (95% CI)
Age at baseline (SD), years	27.4 (6.9)	27.4 (6.9)	
Follow‐up, person‐years	13,305	654,938	
Mean follow‐up (SD), years	11.2 (3.4)	11.0 (3.6)	
Cancer diagnosis at baseline, *n*	5 (0.4%)^*^	308 (0.5%)^*^	0.8 (0.3–2.0)
Diagnosis of gynecomastia, *n*	193 (16.2%)^**^	1034 (1.7%)^**^	9.3 (8.1–10.8)
Diagnosis of infertility, *n*	113 (9.5%)^**^	2997 (5.0%)^**^	1.9 (1.6–2.3)
Diagnosis of testicular dysfunction, *n*	35 (2.9%)^**^	175 (0.3%)^**^	10.0 (7.0–14.3)

Abbreviations: Androgen, anabolic androgenic steroid; CI, confidence interval; SD, standard deviation.

* Cases known at baseline (%).

** Cases known at baseline plus incident cases during follow‐up (%).

The average length of follow‐up was 11.2 (SD = 3.4) years for the androgen users and 11.0 (SD = 3.7) years for the controls. The androgen users were followed for a total of 13,305 person‐years, and the controls were followed for 654,938 person‐years. During this long follow‐up period males using androgens did not receive diagnoses of prostate or breast cancer. Among the controls, 17 cases of prostate cancer were identified, with none exhibiting breast cancer. In total, 13 androgen users and 612 control subjects were diagnosed with any cancer but non‐melanoma skin cancer. This corresponded to an IRR of 1.05 (95% CI: 0.55–1.81; *p* = 0.9) suggesting no significant difference in overall cancer risk between the two groups. The incidences of malignancies detected in androgen users and controls are presented in more detail in Table 2.

**TABLE 2:** Malignancies in 1,189 androgen users versus 59,450 control subjects.

Malignancy type	Incident numbers (95% CI)		Incidence, per 100,000 person‐years (95% CI)		IRR (95% CI)	
	Androgen users	Controls	Androgen users	Controls		*p*‐value
Malignant neoplasm of prostate	0 (0–3.7)	17 (9.9–27.2)	0 (0–27.8)	2.6 (1.5–4.2)	0 (0–11.93)	1
Malignant neoplasm of breast	0 (0–3.7)	0 (0–3.7)	0 (0–27.8)	0 (0–0.6)	NA	NA
All cancers but non‐melanoma skin cancer	13 (6.9–22.2)	612 (564.5–662.5)	98 (52–167)	93 (86–101)	1.05 (0.55–1.81)	0.9

Abbreviations: Androgen, anabolic androgenic steroid; CI, confidence interval; IRR, incidence rate ratio; NA, not available; SD, standard deviation.

**FIGURE 1:**
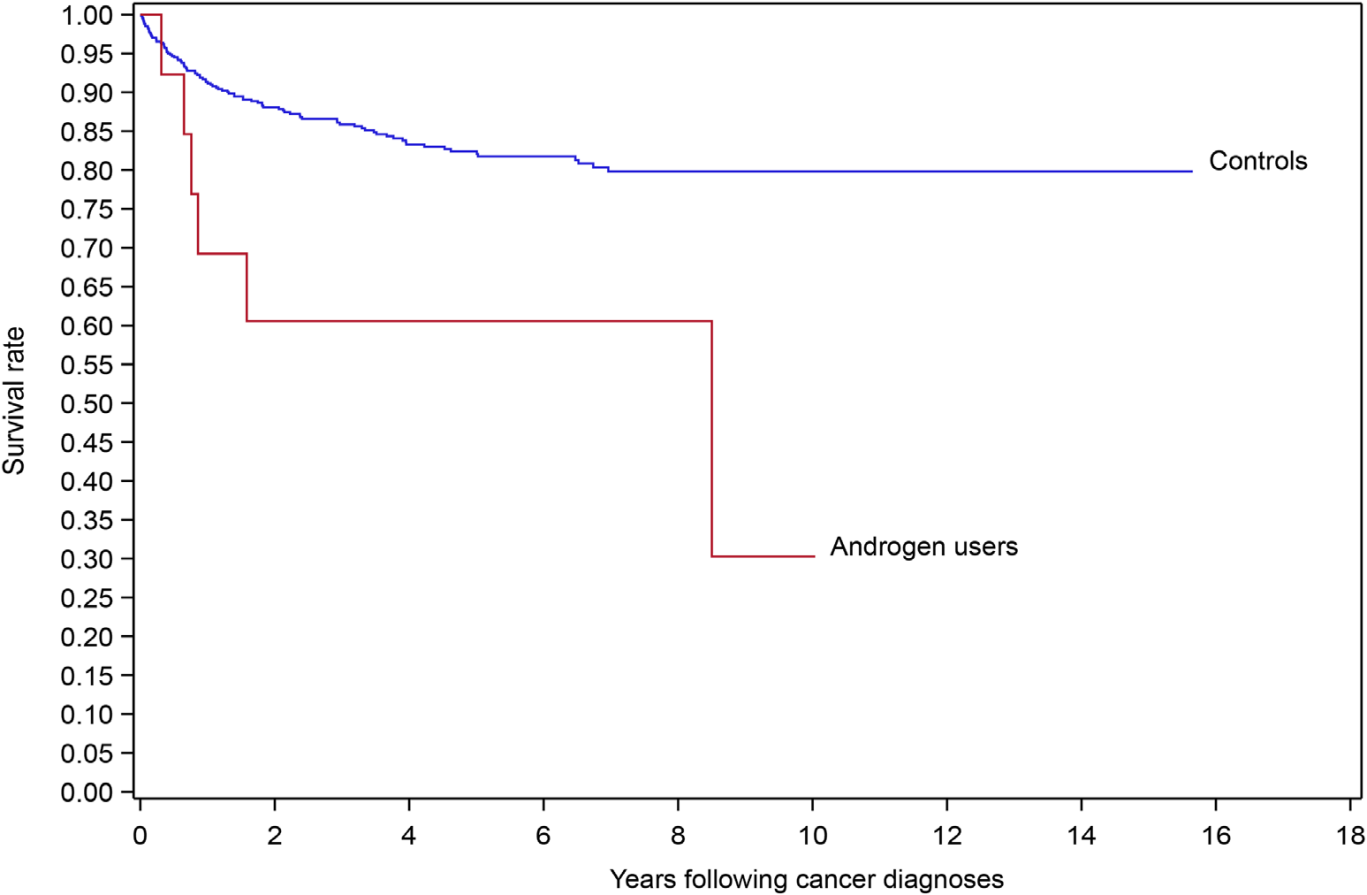
Survival rate following cancer diagnosis was significantly lower among androgen users compared to controls. Hazard ratio of 3.07 (95% CI: 1.35–7.00; *p* = 0.005).

The mortality following the cancer diagnoses among AAS users and controls is shown in Figure 1.

## 4 DISCUSSION

The use of androgens has become a significant global public health concern.^17^ According to a meta‐analysis including 187 studies, the global lifetime prevalence rate of androgen use in males is 6%.^18^

This study investigated the potential association between the use of androgens and the risk of cancer overall and specifically prostate and breast cancer. The results indicate that males using androgens did not face an elevated short‐term risk of cancer, including prostate and breast cancer when compared to a control group. We have previously shown that this cohort of androgen users have a high prevalence of somatic side effects, and by extending our follow‐up with more than 5 years we had expected to detect certain signals with regard to neoplastic disorders.^2^, ^19^, ^20^

While there is a shortage of cohort studies addressing the connection between androgen abuse and cancer risk, there are more studies examining the testosterone therapy at typical physiological doses.^21^, ^22^, ^23^ These studies have not identified an increase in the risk of prostate cancer in men using replacement doses of testosterone.^23^, ^24^, ^25^ Gynecomastia is prevalent among bodybuilders probably due to excessive amount of estradiol, which arises from the metabolism of testosterone. Transgender women, who were assigned male at birth but identify as female treated with estrogen have a 46 times higher risk of developing breast cancer compared to estrogen unexposed *cis*‐men.^26^, ^27^ Furthermore, it has previously been shown that high estradiol concentrations in men is associated with breast cancer.^6^ It should be acknowledged that the metabolic processes and effects of estrogen might vary, depending on whether it is synthesized from androgens or directly ingested.

### 4.1 Strengths and limitations

Several factors should be considered in the interpretation of these results. We were able to follow patients for up to 17.5 years following their doping sanctions. Nevertheless, in the context of cancer, this duration may be regarded as relatively short, especially given the relatively young age of the subjects at enrollment. Both prostate and breast cancer are rare in males under the age of 50 years.^28^, ^29^ This limitation means that the potential long‐term effects of androgen use may not have been fully captured. Additionally, it is important to mention that the participants in the study were identified based on doping sanctions and not confirmed androgen use in all cases. However, since the group that was sanctioned because they refused to participate in the doping control had an identical prevalence of side effects that can be attributed to androgen use, this is deemed to be of minor importance.^2^ Detailed knowledge about the dosing, duration, and subtype of androgens would have been preferable. Additionally, it should be noted that androgen users generally have more hospital visits than control subjects,^2^ and this may lead to more diagnostic work‐up and thereby incidental findings. Given the low incidence of neoplastic disorders in this study, we do not assume that this has caused any systematic bias. There are several other factors associated with an increased cancer risk that could possibly also be linked to androgen use. These factors include diet, alcohol consumption, weight, smoking, concurrent use of multiple drugs, and viral infections.^30^ It would have been strength if we had the opportunity to control for these factors in our study. However, with only a total of 13 androgen users diagnosed with cancer, such corrections would not have had an impact on the conclusion.

The major strength of the current study is the high‐quality registries associated with the Danish Healthcare System, which likely have a sensitivity close to 100% in the case of a cancer diagnosis excluding non‐melanoma skin cancer.^14^, ^16^

## 5 CONCLUSIONS

Our cohort study did not find that androgen users were at an increased risk of cancer overall, and specifically, there were no cases of breast or prostate cancer. This implies that the absolute risk of developing malignancies in androgen abusers is low and approximates that of the general population. However, we cannot exclude androgens as a risk factor for cancer due to the limited sample size in comparison to cancer incidence, the relatively short follow‐up period, and the young age of subjects.

## CONFLICT OF INTEREST STATEMENT

The authors declare no conflicts of interest.

## Data Availability

The data produced and examined during this study are not accessible to the public to ensure the confidentiality of the participants. Upon request, the corresponding author will provide specific information regarding these restrictions and any conditions that may allow for access to certain data.
